# Attitudes & behaviors toward the management of tobacco smoking patients: qualitative study with French primary care physicians

**DOI:** 10.1186/s12875-021-01620-8

**Published:** 2022-01-14

**Authors:** Guillaume Coindard, Michaël Acquadro, Raphaël Chaumont, Benoit Arnould, Philippe Boisnault, Rachel Collignon-Portes, Didier Duhot, François Raineri, Béatrice Tugaut, Henri-Jean Aubin

**Affiliations:** 1 French Society of General Medicine, Issy-les-Moulineaux, France; 2 ICON, Patient-Centered Outcomes, 27, rue de la Villette, 69003 Lyon, France; 3 Université Paris-Saclay, Inserm, CESP, AP-HP, Université Paris Saclay, Villejuif, France

**Keywords:** Tobacco cessation, Primary care physician, Attitude, Behavior, Public health

## Abstract

**Background:**

Smoking cessation is a major public health issue. In France, primary care physicians (PCP) are the first contact points for tobacco management. The objective of this study was to understand how PCPs are involved in the management of smoking cessation: ownership, commitment, barriers.

**Methods:**

A qualitative study was conducted using group and individual semi-structured techniques with PCPs. A thematic analysis of verbatim transcripts was performed to identify concepts and sub-concepts of interest. Saturation was evaluated retrospectively to ensure adequate sample size.

**Results:**

A sample of 35 PCPs were interviewed, 31 in four focus groups and four in individual interviews. PCPs discussed their roles in the management of tobacco smoking cessation, including the different strategies they are using (e.g., Minimal Intervention Strategy, Motivational Interviewing), the multiple barriers encountered (e.g., lack of time, patients’ resistance to medical advice), the support resources and the treatment and intervention they prescribed (e.g. nicotine replacement therapy, supporting therapist).

**Conclusions:**

This study provides a better understanding of the beliefs, attitudes, and behaviors of PCPs in managing smoking cessation. Guiding and encouraging patients toward smoking cessation remains a major objective of PCPs. While PCPs reported that progress has been made in recent years in terms of tools, technology and general awareness, they still face major barriers, some of which could be overcome by appropriate training.

**Supplementary Information:**

The online version contains supplementary material available at 10.1186/s12875-021-01620-8.

## Background

Tobacco smoking is a major public health issue worldwide. Primary care physicians (PCP) are the leading support for patients in tobacco smoking cessation since they are their first healthcare contact [1–4]. They may however experience difficulties providing the appropriate support to their smoking patients. This has been reported worldwide for various reasons, such as lack of time, limited training on smoking cessation and lack of patients motivation to quit [5–7]. As smoking prevalence seems to be recently declining in France [8–10], about half of the smokers who attempted to quit did not seek the input of a health professional; the most often used aids were electronic cigarettes (26.9%), nicotine replacement therapy (18.3%), health professionals’ support (10.4%) and the national smoking cessation helpline service (9.1%) [11].

Evaluation of PCPs’ current practices and providing PCPs with appropriate training are among the missions of the French Society of General Medicine (Société Française de Médecine Générale – SFMG). For a better understanding of French health care service, the authors would like to point out that each patient contracts with a PCP chosen by both sides. Fee-for-service payment remains the classic and dominant method of payment in France, and about 25% of PCPs have academic activities, mainly in the form of hosting medical students in their practices.

The objective of this study was to obtain a better understanding of the factors that influence PCPs’ commitment to managing their patients’ tobacco use. In particular, the identification of PCPs’ attitudes, behaviors, and stated beliefs in tobacco smoking cessation guided our project. Use of the results will be valuable in defining new, targeted, refined, and effective training programs for tobacco smoking cessation in primary care.

### Materials and methods

This article was written in accordance with the standards for reporting qualitative research [12].

This qualitative study was supported by a Steering Committee (SC) convened by SFMG. Composed of experts in tobacco-smoking patients’ management and qualitative research as well as members of SFMG, the SC held meetings at each stage of the research process to ensure the quality, relevance and validity of the study. The role of the SC members was to refine the study objectives, the research methods and to discuss the results. The authors were members of the SC, half of them from the SFMG, the others were tobacco smoking specialist or methodologists.

### Study population

The recruitment of 30 to 40 PCPs was planned for participation in five focus group (FG) discussions. No specific selection criteria were applied to recruit the PCPs. All participants were recruited by SFMG via their network. An invitation was sent by email to PCPs located in and around three target cities: Lille, Paris and Lyon. These cities were chosen as they were representative of areas with respectively high, medium and low rates of tobacco smoking [13]. In addition, to ensure diversity regarding the approaches used for tobacco smoking cessation, the recruitment of four PCP with a strong orientation to smoking cessation management was planned for individual interviews (II). Each one of these four PCPs should cover one of the following specialties, respectively: tobacco addiction, homeopathy, hypnotherapy and acupuncture. The participants were financially compensated for their time.

### Focus group discussions & individual interviews

A dual approach using FG discussions and II was chosen to enhance data collection [14]. As the FG discussion approach is based on a dynamic construct between the participants [15], this was an appropriate method to understand the current systematic professional practices of the PCPs. In addition, II were used to explore the perspectives of PCPs using different practices. The FG discussions and the IIs were conducted in November and December 2019 following a semi-structured interview guide [16]. It was created to detail each theme that needed to be covered during the discussions/interviews. The semi-directive interview technique involves open-ended questions in order to collect spontaneously reported information, while avoiding leading questions. If the PCP did not spontaneously address themes, the interviewer specifically probed these themes, as indicated in the interview guide (Additional file 1). The broad topic areas included:

- Tobacco smoking patients’ management,
- Barriers encountered by PCPs,
- PCP’s role regarding prevention and personal representations,
- PCP’s ease of adaptation to new tools.

Themes and probes of the interview guide were developed based on previous published study findings [6, 7, 17].

Each FG discussion lasted approximately two hours, and each II about one hour. FG discussions and IIs were digitally audio-recorded with participants’ permission, and then transcribed verbatim. Discussions/interviews were conducted by a moderator trained in qualitative interviewing. IIs were conducted over the phone.

### Analysis

The participant sample was described using descriptive statistics. Qualitative analysis of de-identified verbatim transcripts was performed using Atlas.ti software (version 8) [18]. Thematic analysis [16] was used to create codes corresponding to concepts and sub-concepts, as well as coding specific participant quotes. The coding scheme was initially developed based on themes and probes of the interview guide; it was then updated throughout the analysis process according to concepts and sub-concepts that emerged from the discussions/interviews. The concepts and sub-concepts were then reviewed to identify how they are grouped and what were their relationships between them.

Saturation evaluation is a retrospective justification of sample size in qualitative research. Saturation is defined as the point at which no new, concept-relevant insights are likely to be obtained [19]. Saturation can be considered to have been reached for a study when a consistent pattern in participants’ responses is achieved and when no new concepts emerge [20]. Participant responses were analyzed per FG and II in chronological order (the order in which the data was collected). This saturation checking process was used for the global set of data collected, including both FG discussions and IIs.

### Ethical consideration

The present study was conducted in compliance with local ethical principles. The study protocol was submitted and approved by the French National Commission for Data Processing and Privacy (‘Commission Nationale de l’Informatique et des Libertés’) and the French National Medical Association (‘Conseil National de l’Ordre des Médecins’). Each participant received appropriate information note regarding their involvement and rights.

## Results

### Participant sample

Four FG discussions were conducted with a total of 31 PCPs attending. Despite our efforts, it was not possible to recruit a PCP with a specialty in acupuncture. A PCP who was considered for participation in a FG discussion finally ended up being interviewed individually as he/she was not available at the time the FG in his/her area was conducted. Thus, in total, four IIs were conducted. The characteristics of PCPs who participated in the FG discussions and IIs are presented in Table 1.

**Table 1:** Focus group discussions & individual interviews: PCPs characteristics (*n* = 35)

Characteristics		Focus groups *n* = 31	Individual interviews *n* = 4	Total n = 35
Gender (n)	Male / Female	18 / 13	1 / 3	19 / 16
Age (years)	Mean (range)	54 (30–70)	54 (34–62)	54 (30–70)
Location (n)	Urban / Semi-rural	20 / 11	3 / 1	23 / 12
Medical practice (n)	Group	17	3	20
	Solo	11	1	12
	Retired	2	/	2
	Replacement	1	/	1
Medical status (n)	Liberal	27	3	30
	Active retired	2	/	2
	Intern	1	/	1
	Employee	1	1	2
Activity (consultations/year)	< 2500	7	3	10
	2500 à 4500	12	/	12
	4500 à 7000	9	1	10
	> 7000	2	/	2
	Missing data	1	/	1
Academic function (n)	Yes / No	13 / 18	1 / 3	14 / 21
Accepting medical representatives (n)	Yes / No	9 / 22	2 / 2	11 / 24
Other specialty (n)	Hypnotherapy	2	1	3
	Osteopathy	1	/	1
	Homeopathy	/	1	1
	Tobacco addiction	/	1	1
Tobacco smoking status (n)	Never smoked	19	2	21
	Ex-smoker	9	1	10
	Smoker	3	1	4
Tobacco / addiction specialist diploma (n)	Yes / No	2 / 29	1 / 3	3 / 32

*PCP:* Primary care physician

### Qualitative analysis

All PCPs participating in the FG discussions and IIs were interested in the management of tobacco smoking patients theme. PCPs reported that tobacco consumption and addiction was almost never the primary reason for a PCP consultation. Their attitudes regarding tobacco smoking management varied depending on the target population (pediatrics vs. adults vs. elderly) and their specialty. They spontaneously mentioned their own tobacco smoking status, and talked about the advantages and drawbacks of being a smoker, an ex-smoker, or a non-smoker PCP (e.g., understanding addiction, being a good example, etc.). Some themes were controversial between participants, such as electronic cigarettes, or the use of pharmacological treatments for tobacco smoking management. No complete consensus was reached during the FG discussions: depending on the PCPs own theories, experience and beliefs, their practices were mostly different.

The saturation checking process showed that saturation was reached at the first II, while the four FG discussions had been organized first (Additional file 2).

The following sections present the findings from both the FG discussions and the IIs and describe each concept and sub-concept with a number of quotes supporting them. Illustrative quote sample is available in Additional file 3.

#### Role of the PCPs

PCPs acknowledged their role in preventing patients from initiating tobacco smoking (mainly directed at adolescents) and in supporting patients in their cessation process. The act of reiterating advice (37 quotes), asking questions regarding tobacco smoking status or smoking cessation contemplation was part of the role of PCPs. The reiteration in itself was a very important concept to give the opportunity for patients to elaborate, and to signal that the PCP was interested in the patient’s health.

Primary prevention (17 quotes) was very important especially for adolescents, so that they would never initiate tobacco smoking. It was almost always absent for the older adult population or for the elderly.

Supporting patients (15 quotes) was part of PCPs’ role, providing follow-up when patients were interested in quitting, and providing the appropriate support (psychological, pharmacological, and behavioral) depending on the needs. Providing relevant information (14 quotes), medical facts, discussing health risks, and describing opportunities for treatment was part of the PCPs’ role when managing tobacco-smoking patients.

All the PCPs agreed feeling legitimate (9 quotes) talking about tobacco smoking cessation, as they were first-line healthcare practitioners and saw patients very often. A few PCPs thought they were responsible for generating patients’ motivation (4 quotes) for tobacco smoking cessation, whereas most PCPs were not very engaged in doing so, mostly because they did not want to be perceived as confrontational.

#### Target population / groups

PCPs explored tobacco smoking status systematically for each new patient (19 quotes), to document medical records. If a patient was identified as a tobacco smoker, they regularly followed-up on the topic by asking the patient if she/he was still smoking or if she/he had attempted to quit at some point.

If the patient had a major health event that occurred recently (20 quotes*)*, such as a stroke or infarct, PCPs generally used this event as a strong reason to consider tobacco smoking cessation. Patients suffering from a chronic condition (10 quotes) (e.g., cancer, chronic obstructive pulmonary disease) were also a preferred target for PCPs regarding tobacco smoking cessation, and they would usually bring up the topic.

When discussing with adolescents, PCPs would usually try to talk with them in the absence of the parent so that it was easier for them to open up (7 quotes*).* PCPs would use a more casual way of talking, and sometimes be more direct in providing advice.

#### General strategies by PCP

Almost all PCPs were more likely to give general advice or to ask a general question rather than being too intrusive or insistent (28 quotes). Their main strategy was to put themselves in a situation where the patient knew that the PCP was available for them rather than a proactive player insisting on taking action in quitting tobacco smoking.

Being understanding, non-judgmental, and not blaming patients for tobacco smoking (22 quotes) was reported as being one of the most important strategies. Encouraging ambivalent patients and being positive was considered very helpful in developing a therapeutic alliance.

Being opportunistic (13 quotes) was considered a good strategy for PCPs. If patients allowed the opportunity to talk about tobacco smoking cessation, they were ready to jump in and discuss their needs.

Nicotine replacement therapy (10 quotes) was proposed very regularly after a first or second appointment with the PCP as an aid for tobacco smoking cessation.

Adapting to the patients’ personality (8 quotes), understanding their needs and what arguments would be the most helpful was one of the strategies employed by PCPs.

Some PCPs sometimes confronted patients with strong and shocking arguments (7 quotes). This strategy was mostly employed when the PCP felt they had tried everything or were tired of remaining unconditionally supportive.

#### Specific strategies by PCP

Scheduling a dedicated consultation (13 quotes) was a strategy sometimes used by some PCPs who specialized in tobacco smoking cessation or were willing to allow time in a dedicated appointment to discuss the topic in depth. Usually these consultations took from 15 to 45 min depending on PCP’s specialty. A follow-up consultation (10 quotes) was often provided to assess and improve the strategies or treatments provided in a previous consultation.

Help from non-smoking and supportive friends and family (11 quotes) could help toward the goal of tobacco smoking cessation. This strategy depended on the relatives’ and, more broadly, the social network’s characteristics.

Physical activity (10 quotes) was often advised to cope with withdrawal symptoms, or as an argument for primary prevention. It was more effective with adolescents who were willing to have a sports career or for whom sport played an important role.

Money was a well-known argument amongst PCPs, and it could be effective for some patients who were self-conscious about their budget (10 quotes). PCPs considered it to be effective for adolescents.

Some PCPs sought patient involvement (8 quotes) when deciding on their treatments and therapies as a means to strengthen the tobacco smoking cessation engagement.

Progressive reduction of cigarettes (8 quotes) rather that abrupt and complete withdrawal could be an effective strategy for some patients according to PCPs.

Talking about the incompatibility of tobacco smoking with oral contraception (6 quotes) was a good strategy for adolescents and young women to encourage tobacco-smoking cessation.

Talking about the physical disadvantages of tobacco smoking (5 quotes), such as skin color, smell, finger color was also a good strategy for some patients.

Losing liberty (5 quotes) and becoming dependent on tobacco smoking could be another strong argument for receptive adolescents.

Minimal intervention strategy (5 quotes) was employed by most PCPs, but very few correctly named it as a strategy.

PCPs discussed self-help books (4 quotes) for tobacco smoking cessation as a strategy that could work with patients receptive to written guidance.

Impotence risk evocation and fertility (3 quotes) was a strategy seldom used for male patients to encourage them to consider tobacco-smoking cessation.

As an urge to smoke is usually short-lived, delaying a given cigarette (3 quotes) to a later time could help reduce the amount of cigarettes smoked, especially when between-cigarette duration was slowly increasing.

Brief intervention (3 quotes) and Motivational intervention (2 quotes) were strategies that very few PCPs reported using.

#### Identification of relevant factors

To define the most appropriate specific strategy, PCPs first identified relevant factors during their discussions with patients. PCPs tried to identify what forms of dependence (25 quotes) (i.e., physical, psychological, behavioral) their patients experienced to define the appropriate strategies.

PCPs tried to identify which elements could drive their patients’ motivation for change (21 quotes), such as family, own health, or finance, and tried to gauge their motivation level.

Identifying previous cessation attempts (17 quotes) could help to find the appropriate strategy that might work for the patient, while discarding those that did not previously work.

Discussing with the patient what would be their need regarding nicotine replacement therapy (10 quotes) could help reassure the patient and dispel their fears about a specific product.

Identifying the current barriers (6 quotes) (e.g., social environment, dependence, etc.) that prevent a patient from engaging in tobacco smoking cessation could help PCPs to find the appropriate strategy.

Gauging the amount of cigarettes smoked during a day (6 quotes) or on specific events, could help PCPs to determine what would be the relevant motivational levers to act on.

Time of first cigarette of the day (3 quotes) was sometimes asked to get a sense of the patient’s ritual and habits regarding tobacco smoking.

#### Barriers to action from PCP

Lack of time (13 quotes) was reported as being the greatest barrier to properly discussing tobacco-smoking cessation with all patients.

Patients with a high socioeconomic status (12 quotes) could be resistant to PCP’s authority and it might be difficult to convince them to quit tobacco smoking, while patients with a low socioeconomic status might be more receptive to argumentation, although their social condition might impede their efforts toward tobacco smoking cessation.

Social environment (12 quotes) could play a significant role in the difficulties quitting tobacco smoking, especially if the patients’ acquaintances or relatives are tobacco smokers.

Weight gain (10 quotes) after tobacco smoking cessation if no physical activity was planned could be a strong deterrent to stop. Indeed, patients often fear withdrawal symptoms (10 quotes).

A co-occurring addiction (10 quotes) might often delay tobacco-smoking cessation, especially if the competing addiction is considered more serious for the patients’ health than tobacco smoking.

Some patients (particularly adolescents and young adults) might experience a feeling of invulnerability (8 quotes), having not experienced/witnessed health problems despite their tobacco smoking behavior, or an acquaintance’s tobacco smoking behavior.

PCPs might sometimes consider tobacco smoking a matter of personal lifestyle choice (7 quotes) and patients might consider their intervention too intrusive.

Patients might have competing priorities (6 quotes) beside tobacco smoking cessation, and sometimes tobacco smoking might help coping with stressful events.

PCPs identified marketing within the tobacco industry (5 quotes) as a major antagonist toward tobacco smoking cessation.

Patients sometimes refused (5 quotes) to relate their health condition to their tobacco smoking behavior.

On the one hand, a tobacco smoking PCP could be viewed as illegitimate (5 quotes) to provide tobacco smoking cessation advices as he/she lacks credibility. On the other hand, non-tobacco smoking PCP could be viewed as illegitimate (4 quotes) to provide tobacco smoking cessation advices as he/she lacks the experience of tobacco smoking and struggling with an addiction.

Patients might feel overconfident (3 quotes) regarding their ability to quit anytime, underestimating their substance dependence, which is sometimes difficult for the PCP to deal with.

PCPs sometimes reported lacking skills (3 quotes) regarding the appropriate way to support a patient, and the use of all cessation strategies.

Mostly PCPs in IIs admitted that they feared jeopardizing the therapeutic alliance (3 quotes) by being too insistent with their advice and questions.

Some PCPs reported that it was very challenging for psychiatric patients (3 quotes) to stop tobacco smoking.

Some PCPs reported that it was challenging to catch adolescent (3 quotes) patients as they have limited opportunities to consult with adolescents, except for contraception or sport certificates.

#### Resources

PCPs identified other healthcare professionals (25 quotes) as their main resources when considering tobacco smoking patient management. Pharmacists, smoking cessation specialists, nurses, psychologists could be useful resources.

Websites and smartphone applications (22 quotes) could be useful resources for PCPs as well, and some PCPs even wrote down web resources on their patients’ prescription to help them seek assistance.

Having the personal experience of tobacco smoking cessation (12 quotes) could be helpful for PCPs to understand their patients when they were facing difficulties following a program. It might be used as an argument depending on the patient-PCP relationship.

Training courses (10 quotes) could help PCPs maintain awareness of new strategies, keeping them up-to-date when it came to giving advice and providing relevant strategies to patients.

Patients’ motivation (9 quotes) was often considered as a strong prerequisite to even consider planning a tobacco smoking cessation attempt. If it was absent, PCPs usually waited for patients’ motivation to emerge.

Pregnancy (9 quotes) was considered as a key moment for PCPs to help new mothers maintain their non-tobacco smoker status, as most of them usually quit tobacco smoking on their own when they get pregnant.

Recent tobacco smoking denormalization (9 quotes) was considered useful by the PCPs as it helped the collective mind to stop smoking.

The full reimbursement (8 quotes) of most nicotine replacement therapy in France has a positive impact when proposing this strategy for physical dependence.

Events such as “Stoptober” [21] (7 quotes) are considered helpful by most PCPs since they provide an additional opportunity to address tobacco smoking cessation.

The price increase of cigarettes (5 quotes) could be a strong argument to stop tobacco smoking, and PCPs often quoted the role of the government in this matter. They also quoted the situation in Australia where cigarettes are very expensive.

Flyers and posters could be helpful materials (5 quotes) to display in the waiting room. Some PCPs reported having less access to such materials.

PCPs discussed the fact that medical research progress provides (3 quotes) relevant additional arguments to submit to resistant patients.

Some assessment tools (3 quotes) such as Fagerström Test for Nicotine Dependence, or carbon monoxide testing devices could provide an objective assessment of the outcome to patients with positive feedback, as reported by the PCPs.

Some PCPs mentioned that being aware of child passive tobacco smoking (3 quotes) consequences could help tobacco-smoking parents considering cessation.

#### Treatment and interventions

PCPs also discussed various treatments and interventions during FGs and IIs. The number of PCPs that reported a habit of prescription is detailed in Table 2.

**Table 2:** Number of PCPs (*n* = 35) reporting a habit of prescription of treatment and/or supporting therapy

Habit of prescription	PCPs (n)
*Treatment:*	
Nicotine patch	15
Electronic cigarette	6
Nicotine gum	5
Pharmacological treatment	4
Nicotine spray	1
*Supporting therapy:*	
Hypnotherapy	9
Psychological therapy	7
Acupuncture	7
Auriculotherapy	5
Tobacco specialist	5
Homeopathy	4
Addiction specialist	3

*PCP:* Primary care physician

Nicotine patches were the most common nicotine replacement therapy prescribed and were used by all PCPs. Some PCPs encouraged the use of electronic cigarettes to cope with physical dependence, but some considered electronic cigarettes harmful as it maintained behavioral dependence, as well as pharmaco-dependence depending on nicotine dosage. Nicotine gums were usually prescribed in combination with patches to cope with urges, but presented also the drawback of overconsumption in some cases. Pharmacological treatments were rarely prescribed, and when they were it was usually as a second- or third-line option. Varenicline was quoted (*n* = 7) as harmful, with depression and suicide mentioned as serious adverse events. Last, nicotine spray was mentioned a couple of times but only one PCP prescribed it.

Some PCPs were trained in hypnotherapy and used it regularly in their clinical practice, especially regarding tobacco smoking cessation. Those not trained usually referred to other hypnotherapists they trusted. Psychologists were referred by PCPs as helpful for managing psychological dependence for patients who could afford it. Acupuncture, auriculotherapy and homeopathy were advised by some PCPs if this option was deemed effective by their patients, even if the PCP was doubtful of the scientific grounds for the therapy. Tobacco specialists were usually referred when PCPs talked about the ‘Tabac Info Service’ (French smoking cessation helpline). Otherwise, some PCPs felt that tobacco specialists did not add any value to tobacco smoking cessation. Addiction specialists were usually referred when there were co-occurring dependencies, or a dependence that was more severe.

## Discussion

This qualitative study identified a comprehensive list of determinants encountered by PCPs in their routine practice when managing tobacco-smoking patients. To perform their roles, PCPs use general strategies and prevention interventions when interacting with patients, as well as specific strategies informed by their identification efforts when interacting with patients. PCPs encounter different barriers when performing their strategies, either on the patients’ side, or on their own side. External resources are available to PCPs and positively influence their intervention strategies, as well as the receptiveness of target patients.

Not all studies have reported that PCPs’ considered that proposing smoking cessation advice or intervention was fully legitimate. For example, a Dutch qualitative study reported that, not perceiving smoking as an illness, PCPs proactively advised smoking cessation only if the patients developed a smoking related illness. In any case, the behavioral support would be the responsibility of other professionals. In this study, PCPs refused to be involved in prevention tasks and health promotion, and thus did not confront their “healthy” patients with their smoking behavior [17]. Another study reported what we could qualify an ambivalent attitude among PCPs, with a global feeling of lack of legitimacy on the one hand, and a clear awareness to have a role to play on the other, when it comes to addressing the issue of smoking cessation [22]. The impact of the occurrence of a smoking-related medical condition on the repetition of the smoking status inquiry, the advice to quit smoking, and eventually a smoking cessation intervention has consistently been reported in other studies performed in PCP settings [17, 22, 23].

Our finding that PCPs believe the motivation to quit smoking must above all come from the patient is consistent with other reports [17, 22]; in one study PCPs considered furthermore that tobacco smoking cessation is the patients’ responsibility [17].

The inclination not to be too intrusive – in order to avoid jeopardizing the therapeutic alliance – found in our study is in contrast with the results of a Dutch qualitative study, where PCPs did not fear jeopardizing the doctor-patient relationship by discussing smoking, even though they did not want to patronize their patients [17]. Another study stressed the importance of the therapeutic alliance in the PCPs relationship with their patients when they considered a smoking cessation intervention [22].

The sampling procedure might be a source of limitations to our study: we enrolled volunteers from a pre-specified subset of city areas. First, we have not covered rural areas – now a minority population in an highly urbanized French population – in which the first priority is the actual access to Primary Care due to poor geographical coverage. Second, only three areas were selected, and we did not apply a sampling procedure for this selection; however, these three areas were chosen to present a good coverage of important variations in terms of epidemiology [13]. Third, only volunteers were enrolled, which obviously favored the participation of motivated, interested, and available PCPs. However, while this may lead to missing the reasons for which French PCPs may be not motivated or interested in tobacco cessation, we believe it does not affect our research findings and conclusions, as motivated and interested participants were best placed to “understand how PCPs are involved in the management of tobacco smoking cessation: ownership, commitment, barriers”. Furthermore, interest and motivation does not necessarily imply different representations: for example, even if some PCPs were not able to participate due to their overload and lack of time, the time limitation emerged as a major barrier among the study participants. The participants sample was diverse in terms of age, gender, location, medical practice. The distribution between non-tobacco smokers, formerly-tobacco smokers and tobacco smokers was balanced and representative of the real world proportion [24]. In addition, the three IIs conducted with PCPs with a specialty (i.e. hypnotherapy, homeopathy and tobacco specialty) reported similar concepts to those from the FG discussions. This suggest that our findings are comprehensive, consistent, and robust.

Our study does not quantify the proportion of PCPs who would support each of the themes and the views reported during our qualitative work. Nor does it allow a quantitative comparison in importance or frequency. This limitation is inherent to qualitative research and does not invalidate our findings, which are based on in-depth discussions and confirmed by clear saturation. Beyond the clear picture they give of the situation today in France, our findings and the methods could support the development of surveys or psychometric tools that would allow individual characterization of PCP’s profile regarding the management of tobacco smoking patients.

Our study was conducted in France, and the findings cannot be generalized to other countries, mainly because they are context-dependent and because of cultural specificity. However, the method is replicable and other country-specific studies based on same approach would greatly allow to inform the field about the invariants and the variants in the role of PCPs in the management of consumption of and addiction to tobacco smoking.

All PCPs felt legitimate in their preventive role, even if they considered it to be time consuming and difficult. To fulfil this mission, they considered that they must often repeat over and over again the same information, provide education on smoking behavior and cessation, and offer regular follow-up support to receptive patients. Most PCPs systematically identified smoking status when meeting new patients and creating their medical record. PCPs systematically explore smoking status along with other lifestyle health factors. PCPs reported that they often follow-up about smoking status with patients identified as smokers, especially when another medical condition emerges. PCPs reported that a major / secondary medical event (e.g., stroke, chronic bronchitis, cancer, etc.) was an opportunity to talk again about smoking status or tobacco cessation strategies, as patients are often motivated to take action in light of their new medical condition. However, some patients with severe medical conditions or comorbidities were reported to be resistant to change despite their medical status. PCPs felt they had an important preventive role with adolescents, although considered the task difficult to accomplish because encounters with younger patients were uncommon. However, some opportunities occur when prescribing contraception to young women (e.g., incompatibility of smoking status and a range of contraceptive treatments) or delivering medical certificates for practising sports (e.g., relationship between negative physical performance and smoking status) [25]. PCPs also found beneficial effects for pregnant women as most cease smoking on their own during pregnancy. All PCPs were ready to provide support and advice to pregnant women struggling with smoking cessation [26].

PCPs reported a number of communication strategies (general and specific) with patients. The primary attitude of French PCPs is to remain available to the patient without being too intrusive. Most PCPs were aware of – and many actually reported using – the Minimal Intervention strategy [27]. This strategy was developed to give an opportunity to the patient to reflect upon a possible change in behavior (i.e., “Do you smoke?”, “Do you intend to stop smoking?”). French PCPs believe that the motivation must above all come from the patient, and that being too intrusive would jeopardize the therapeutic alliance with the patient. It has been suggested that this representation, at least for French PCPs, stems from a cultural interpretation of the frontier between respect for the patient’s lifestyle and medical responsibilities [28]. PCPs in France feel that they do not have the right to be too intrusive or insistent, and this may result in a “passive” stance until the patient expresses a will to quit tobacco smoking. As the lack of proactive intervention in primary care has also been reported in other countries, it usually reflects a resistance to preventive tasks rather than the fear of jeopardizing the doctor-patient relationship [17]. Many PCPs declared to prescribe nicotine replacement therapy for all patients willing to stop smoking, nicotine patch being the most prescribed. PCPs reported that since nicotine replacement therapy benefits from reimbursement from the French healthcare system (effective from 1st January 2019), it is easier to prescribe to all, and especially to those with a lower income. PCPs were aware that they could refer their patients to other healthcare practitioners for the management of tobacco smoking cessation (e.g., hypnotherapy, cognitive-based therapy, acupuncture, etc.) and some PCPs undertook training in this respect to address their patients’ need. Despite PCPs measured opinion regarding scientific grounds or benefits of aforementioned available therapies, they were supportive of their patients’ initiative to seek additional support for smoking cessation, as long as they reached for certified professionals.

PCPs reported encountering many barriers in their role of smoking cessation counseling. As in other countries, lack of time for a proper tobacco cessation intervention was paramount. Some PCPs discussed the fact that tobacco smoking cessation was almost never the primary motive of a consultation; hence, there was little time to raise the tobacco smoking issue systematically if the main reason for consultation was not related to smoking. On the other hand, some PCPs acknowledged that a range of tools were available for smoking cessation, that were not necessarily time-consuming (e.g., Minimal Intervention, advising of the tobacco smoking cessation helpline, referring to a mobile application). Patient’s socio-professional category was reported as being a possible barrier. Patients with higher socioeconomic status were considered more likely to be resistant to PCPs’ authority and therefore arguing for smoking cessation was more difficult, while patients with lower socioeconomic status may be more receptive to PCPs’ authority, but are more prone to suffer from an adverse smoking environment impeding efforts toward tobacco smoking cessation [29]. Another significant barrier to tobacco smoking cessation was post-cessation weight gain concerns [30, 31]. These concerns were reported as difficult to challenge, one useful strategy being to encourage physical activity. Such a strategy was not always reported to be effective, as it required harnessing additional motivation from patients to sustain regular training. For some PCPs, the outcomes obtained regarding smoking patients’ management were not always up to their investment in terms of time and effort. This could lead to frustration, disenchantment and eventually to PCPs engaging in a confrontational style in order to elicit a reaction from patients.

Some PCPs were not fully aware of effective interventions and treatments other than nicotine replacement therapy. Most French PCPs expressed an image of varenicline as being unsafe (e.g., depression, increased cardio-vascular risks), despite recent research demonstrating otherwise [32, 33], which is correlated to their prescription habit [32]. Not all PCPs were aware of the recent development of digital support to help smoking cessation [34]. This knowledge was more common amongst younger PCPs who knew about smartphone applications and their effectiveness, even without direct involvement of PCPs [35]. Older PCPs reported witnessing a shift in the general French population’s depiction of tobacco smokers. While in the past, smoking connoted a range of mostly positive and desirable attributes, thus being a widely accepted behavior, exponential escalation of news about tobacco adverse health effects has led tobacco smoking to become routinely depicted in everyday discourse and media representations in a variety of overwhelmingly negative ways [36, 37]. This strong shift toward tobacco smoking denormalization was reported as being helpful by all PCPs in their smoking cessation-counseling role.

This study was the subject of another publication in the form of a university medical thesis [38]. It shows with an inductive analysis by grounded theory that behind the difficulty of this management lies a dual ambivalence, that of the patient and that of the PCP. We know the ambivalences of the dependent patient, less so those of the PCP. Should PCPs generate motivation or just increase it? Should they systematically consider smoking as a public health problem or should they respect the personal liberty and autonomy of the patient (in particular respect for pleasure when the patient mentions it)?

All of this data linked to this study should make it possible to modify French PCPs’ training programs on tobacco smoking cessation. The transtheoretical model of change [39] is not universally included in the training of young French PCPs. We believe it would be relevant to incorporate it into tobacco management training, both to explain patient behaviors and to reflect on the PCPs’ own ambivalence. PCPs would benefit from knowing more about the populations that are more easily accessible to tobacco smoking cessation and providing better support to others. This requires them to be in tune with their own perceptions of tobacco use in order to provide clear, accurate and appropriate information.

## Conclusions

This qualitative study identified a comprehensive list of determinants encountered by French PCPs in their routine practice when managing tobacco-smoking patients. The qualitative data extracted from the FGs and IIs helped to understand the dynamics of the attitude, beliefs and behavior of PCPs regarding their role, general and specific strategies, representation and identification of smokers, barriers and resources in tobacco-smoking patients’ management. The most reported global strategies by PCPs were providing general support and advice regarding smoking cessation without being too intrusive, and having an understanding and non-judgmental attitude with their patients. Secondly, scheduling dedicated and follow-up consultations were specific strategies used by PCPs to allow dedicated time to patients willing to stop smoking. The lack of time to dedicate to tobacco cessation was the most consistently reported barrier by PCPs across all interviews, followed by patients’ social environment. Overall, these key findings will help to better design training programs for PCPs.

## Supplementary Information

**Additional file 1.** Interview guide.

**Additional file 2.** Saturation.

**Additional file 3.** Illustrative quotes.

## Data Availability

The datasets used and/or analyzed during the current study are available from the corresponding author on reasonable request.

## Abbreviations

FG: Focus Group
II: Individual Interview
PCP: Primary care physician
SC: Steering committee
SFMG: French Society of General Medicine (Société Française de Médecine Générale)
