# Supplementary material for: Attitudes & behaviors toward the management of tobacco smoking patients: qualitative study with French primary care physicians
Source: BMC Prim Care. 2022 Jan 14;23:10. doi: 10.1186/s12875-021-01620-8 (PMC8759174; doi:10.1186/s12875-021-01620-8)
Supplement: Supplementary file 1 — Additional file 1. Interview guide. [file 12875_2021_1620_MOESM1_ESM.docx]

## Additional file 1 – Interview guide

| **Starting instructions** |
| --- |
| Please introduce yourself by first name and then tell us your age, place of practice and since when you have been practicing as a doctor.  Briefly describe your experience dealing with smoking patients in general. |
| **THEMES TO PROBE AND EXAMPLES OF QUESTIONS TO ASK** |
| **Theme 1: Management of tobacco smoking patients** |
| - Types of patients with whom doctors take the initiative to talk about tobacco smoking.   - - For the mentioned patients:       - For what reasons do you bring up the subject of tobacco smoking?       - Are the reasons always the same?       - Is it systematic?     - For other patients:       - Do you ever bring up the subject with other patients?       - If so, why?       - If not, for what reasons? - Strategy used when managing of a tobacco smoking patient.   - - Minimal Intervention Strategy: Individual efforts to quit tobacco smoking without the continued assistance of healthcare professionals or organizations, but by (1) devising their own way to quit; (2) receiving brief instructions or advices on how to quit and then quit; or, (3) using a self-help aid or guide to stop     - Motivational Interviewing technique / Motivational Interviewing: an approach which tries to get an individual out of a state of indecision or uncertainty and to motivate him to make positive decisions and to achieve the set objectives.     - Very Brief Advice: Simple advice that is designed to be used in under 30 seconds in almost any situation with a tobacco smoker and is based on 3 elements: establishing tobacco smoker status, giving advice on how to quit and offering to help if needed.     - Frequency of reminder / setting of short or medium term objectives - Therapeutic care: what are their prescriptions? What for?   - - Pharmacological treatment (nicotine replacement therapy, varenicline, bupropion     - Electronic cigarettes     - Other therapies: Cognitive-Behavioralist Therapies (CBT), acupuncture, auriculotherapy, hypnosis. |
| **Theme 2: Barriers** |
| - Barriers encountered when dealing with patients   - - Personal barriers:       - Credibility ("I smoke")       - Legitimacy: "not comfortable meddling in my patients' lifestyle choices" or "I've never smoked, so I don't understand the difficulty of quitting"       - Insurance: "fear of failure - I don't want to burn myself out on things that don't work"       - Communication: "fear of compromising the relationship with the patient"     - Barriers during consultation       - Other priorities for this patient       - Patient objections       - Failure of one or more previous attempts: suffering previously encountered and fears of another failure       - Communication difficulties       - No / little impact of arguments     - Organizational barriers       - Consultation time       - Helping staff for the consultation       - Educational, informative material     - Barriers linked to the stakeholders in tobacco smoking / pharmacological cessation (nicotine substitution, varenicline, bupropion       - Does not know the national / local stakeholders supporting tobacco smoking cessation       - Difficulties with pharmacological treatments (nicotine replacement, varenicline, bupropion       - Difficulties related to electronic cigarettes - Difficulties with certain patient populations in particular   - - Adolescents (including actions that have already worked in the case of adolescents)     - Patients of modest social origin     - Pregnant women |
| **Theme 3: Role of the doctor in prevention and personal representations** |
| - Opinion on the role of prevention in clinical practice - Tobacco smoking prevention mission   - - - Inform about the risks       - Motivate to stop / decrease       - Propose solutions       - Repeat - Means to properly play their role in tobacco smoking management   - - If so, which ones? - Tobacco smoking status: Do you smoke, have you been a smoker, or have you or have you had smokers in your surroundings?   - - To what extent does this experience influence you in the tobacco smoking management of your patients? |
| **Theme 4: Doctor's ability to adapt to new tools** |
| - In the case of monitoring tobacco smoking patients, do you use specific technologies?   - - Call / SMS / Email / Whatsapp / Other apps - How comfortable are you with using new digital tools? - Do you think that new applications could help you in your prevention and monitoring work? |

**Thank you for your participation**
