# Supplementary material for: Attitudes & behaviors toward the management of tobacco smoking patients: qualitative study with French primary care physicians
Source: BMC Prim Care. 2022 Jan 14;23:10. doi: 10.1186/s12875-021-01620-8 (PMC8759174; doi:10.1186/s12875-021-01620-8)
Supplement: Supplementary file 2 — Additional file 2. Saturation. [file 12875_2021_1620_MOESM2_ESM.docx]

BL: Baseline, no focus group conducted; FG: Focus group; II: Individual interview

Saturation was evaluated based on 95 concepts coded on verbatim transcripts from focus groups (FG) discussions and individual interviews (II) that included a total of 35 primary care physicians. FG and II were classified by chronological order.

The first FG (FG01) enabled to identify 76% of the total concepts (72 out of 95 concepts); the second FG (FG02) enabled to identify 91% of the total concepts (86 out of 95 concepts); all the concepts were identified a by the first II (II01); no new concepts were identified by the 3 last IIs.
