# Supplementary material for: Attitudes & behaviors toward the management of tobacco smoking patients: qualitative study with French primary care physicians
Source: BMC Prim Care. 2022 Jan 14;23:10. doi: 10.1186/s12875-021-01620-8 (PMC8759174; doi:10.1186/s12875-021-01620-8)
Supplement: Supplementary file 3 — Additional file 3. Illustrative quotes. [file 12875_2021_1620_MOESM3_ESM.docx]

**Additional file 3 - Illustrative quotes**

| Theme | Concept | Total number of quotes | Example of quote | Participant ID | Participant Characteristics |
| --- | --- | --- | --- | --- | --- |
| Role of PCPs | Reiterating advice | 37 | “We don’t give up on the topic [of quitting smoking]. We write it down carefully in the patient’s file [to talk about quitting smoking at their next visit]. I write it down it a way that I can easily see it.” | PCP24-FG03 | M, 59 yo, NS |
| Role of PCPs | Primary prevention | 17 | “Primary or secondary prevention nonetheless means taking into account a whole age group that begins in childhood. I mean, a child who has asthma at 8 years old needs to remember in his mind that starting to smoke at 15 would be a stupid mistake.” | PCP01-FG01 | M, 60 yo, S |
| Role of PCPs | Supporting patients | 15 | “We support them, we have knowledge, we can tell them about scientific facts that they sometimes don’t know, because sometimes they are very well informed, but not all of them. We can explain to them how a [nicotine] patch could help, with what symptoms. And afterwards, we work with them, if they want, on a plan [to stop smoking] and on top of that, support them [with that plan].” | PCP25-FG03 | F, 58 yo, NS |
| Role of PCPs | Providing relevant information | 14 | “We are doctors. We give information. We say what we know.” | PCP27-FG04 | M, 69 yo, FS |
| Role of PCPs | Feeling legitimate | 9 | “Moderator: And do you, as primary care physicians, feel in fact legitimate to support patients who are trying to stop smoking? Is this your role? Participant: Yes! Much more than a tobacco specialist!” | PCP13-FG02 | M, 60 yo, FS |
| Role of PCPs | Generating patient motivation | 4 | “I think that the doctor’s role is to slowly, one step at a time, help the patient find motivation [to stop smoking].” | PCP06-FG01 | M, 55 yo, NS |
| Target population / groups | Systematic, each new patient | 19 | “The topic of the patient’s lifestyle is systematically discussed with each new patient. So: alcohol, drugs, tobacco, violence.” | PCP04-FG01 | F, 55 yo, FS |
| Target population / groups | After major health event | 20 | “For example, someone who has just had a coronary episode or a heart attack, in general they need to stop [smoking] when they come to see us. They are so afraid that they quit overnight.” | PCP32-II01 | M, 57 yo, FS |
| Target population / groups | Patient with chronic condition | 10 | “The patients with COPD, the asthmatic ones too, I’ll ask them about it [smoking] again and again, all the time […] and [also] all of those [patients] who have risk factors for cardiovascular diseases who come in for a prescription renewal.” | PCP12-FG02 | F, 33 yo, NS |
| Target population / groups | Adolescents | 10 | “I try for a moment each time [I see them] to at least ask the parents if they would be OK to leave us alone. Often, I just put aside a few minutes to talk alone [with the teen]. And, then, I systematically ask them about tobacco, drugs, sexual assault, harassment, and physical and verbal violence at school.” | PCP12-FG02 | F, 33 yo, NS |
| General strategies by PCP | Giving general advice, not being too intrusive/insistent | 28 | “So, the first time I just try to bring up the idea and if the person is open to it, we talk about it a bit more. If she isn’t, then I say we’ll talk about it at the next appointment.” | PCP33-II02 | F, 34 yo, S |
| General strategies by PCP | Being understanding, non-judgmental, and not blaming | 22 | “I think the most important thing is to not make people feel guilty, because in any case if we make them feel guilty that doesn’t work at all; they will become stubborn.” | PCP33-II02 | F, 34 yo, S |
| General strategies by PCP | Being opportunistic | 13 | “There are very few chances for a doctor to talk to a patient about quitting smoking. If a patient mentions the topic, then you need to respond immediately; there’s no magic way of bringing the topic up.” | PCP01-FG01 | M, 60 yo, S |
| General strategies by PCP | Proposing nicotine replacement therapy | 10 | “I’m more inclined to use replacement therapy first because of the results it’s shown in opiate addicts. But it’s true that it’s important to insist that nicotine replacement therapy be well done and of sufficient duration, because it’s hard to stop tobacco but easier to quit [nicotine] patches.” | PCP13-FG02 | M, 60 yo, FS |
| General strategies by PCP | Adapting to patients’ personality | 8 | “So let’s say that for those patients who are more inclined to respond to a visual image, we will try to find something like that for them. [On the other hand], there is no point to talk to someone who responds to words and sounds with visual images” | PCP35-II04 | F, 62 yo, FS |
| General strategies by PCP | Using strong and shocking arguments | 7 | “It may be a bit violent but I think that sometimes we need to say these things. Because sometimes patients become aware [of the risks] and something clicks into place for them.” | PCP27-FG04 | M, 69 yo, FS |
| Specific strategies by PCP | Scheduling a dedicated consultation | 13 | “If the person talks to me spontaneously [about smoking] while they are [at the appointment] for other reasons, I will maybe say: let’s have another appointment on such-and-such a day because we need a little more time and I will explain to you [then] the in’s and out’s of everything.” | PCP32-II01 | M, 57 yo, FS |
| Specific strategies by PCP | Providing follow-up consultation | 10 | “I always plan for an appointment after the person quits […] a few days after they quit and possibly a month afterwards [too].” | PCP13-FG02 | M, 60 yo, FS |
| Specific strategies by PCP | Supporting help from non-smoking and supportive friends and family | 11 | “I say [to the patient], “Talk about it [your goal to quit smoking] to all the people who will help you.” Because there are many people that are happy that their children, their close friends and family [will try to stop smoking] […] and you need to let them know.” | PCP25-FG03 | F, 58 yo, NS |
| Specific strategies by PCP | Advising physical activity | 10 | “So, the behavior aspect of addiction can be treated by using exercise.” | PCP20-FG03 | F, 52 yo, FS |
| Specific strategies by PCP | Using financial argument | 10 | “All arguments are good arguments to stop smoking. Indeed, it’s true that for students, the price of tobacco is a great [argument]. It [tobacco] is very expensive.” | PCP19-FG03 | F, 47 yo, NS |
| Specific strategies by PCP | Seeking patient involvement | 8 | “We need to get the patient involved when we think of strategies [for quitting smoking]. Not everything can come from other people. So it’s together that we can … we [doctors] are available to help him or her but we must also ask him or her to do things so that it works.” | PCP06-FG01 | M, 55 yo, NS |
| Specific strategies by PCP | Reducing progressively cigarette consumption | 8 | “Which cigarettes are the most important ones and which are the least important ones? And try to reduce the less important ones and when they [the patients] are somewhat more motivated, we can talk about it again.” | PCP11-FG02 | M, 36 yo, NS |
| Specific strategies by PCP | Talking about incompatibility of tobacco smoking with oral contraception | 6 | “A convincing argument is the contraceptive pill. I tell them [the patients]: look at it [the pill] and you’ll see, all of the pills are progesterone-based and are contra-indicated when you smoke, even if you smoke e-cigarettes. […] It doesn’t necessarily make them stop but I tell them, I can’t prescribe you the pill because I would be at fault.” | PCP32-II01 | M, 57 yo, FS |
| Specific strategies by PCP | Talking about the physical disadvantages of tobacco smoking | 5 | “When it comes to girls, I convince them more easily [to quit smoking], I tell them it will give them wrinkles, [poor] skin, [bad] teeth” | PCP21-FG03 | F, 47 yo, NS |
| Specific strategies by PCP | Talking about losing liberty | 5 | “They [teenagers] don’t want to lose their freedom. [But in the end,] they are not free because they have been completely turned into little consumers who have lost all freedom.” | PCP08-FG01 | M, 64 yo, NS |
| Specific strategies by PCP | Using minimal intervention strategy | 5 | “So, there’s that classic little question “Do you smoke and do you have the intention to quit smoking?” So, we know that even just asking these two questions can lead to 5% of people trying to stop tobacco. So, I ask that question systematically.” | PCP34-II03 | F, 59 yo, NS |
| Specific strategies by PCP | Discussing self-help books | 4 | “So, there is also a book by Allen Carr that I have them read. [After reading it], practically 8 out of 10 [of the patients], they stop [smoking].” | PCP35-II04 | F, 62 yo, FS |
| Specific strategies by PCP | Discussing impotence and fertility risk | 3 | “I talk about the coronary arteries, the small arteries of the penis, and I let them think, and then I talk about the small arteries of the fingertips. […] And then, after a month they will say “Huh, the penis? Really?” “Of course, yes,” [I will say], “it [smoking] makes you impotent.” And then, bingo!” | PCP04-II01 | F, 55 yo, FS |
| Specific strategies by PCP | Delaying a given cigarette | 3 | “I want to smoke a cigarette, so, I have a conditioned reflex, I take my cigarette. If he waits three minutes before smoking, huh, after three minute, I’m not thinking about [smoking] anymore. It passes. So, after 15 minutes, I’ll be back to thinking about [smoking]. But I won 15 minutes […] After, as he learns, he will tell himself: “Why not wait half an hour, in half an hour I’ll smoke my cigarette.” And so, this type of pedagogy the person learns himself, it’s constructive. It builds his ability to improve himself bit by bit.” | PCP31-FG04 | M, 69 yo, NS |
| Specific strategies by PCP | Using brief intervention technique | 3 | “I’ll bring up the topic just to know, yes or no, does the patient smoke. It’s a simple question, it can’t offend him. And then, I will do a brief intervention” | PCP19-FG03 | F, 47 yo, NS |
| Specific strategies by PCP | Using motivational intervention technique | 2 | “I try to encourage the positive motivations of the patient.” | PCP13-FG02 | M, 60 yo, FS |
| Identification of relevant factors | Forms of dependence (physical, psychological, behavioral) | 25 | “The more dependent they [the patient] is on [tobacco], the more nicotine replacement therapy becomes necessary. Then you have to evaluate [their dependence]. That’s why I say that at the beginning, you need to understand their addiction, you need to understand their relationship [with cigarettes].” | PCP06-FG01 | M, 55 yo, NS |
| Identification of relevant factors | Elements that could drive patients’ motivation | 21 | “How motivated is the patient? Why is the patient motivated? Is it that his grand-uncle just died of an illness, of COPD, of lung cancer? Is it because he ears about it [smoking] again and again? Is it because it [smoking] costs him too much? In the end, well, there’s a whole list of possible motivations.” | PCP13-FG02 | M, 60 yo, FS |
| Identification of relevant factors | Previous cessation attempts | 17 | “If he quit, how did he stop? How did he feel? If he started smoking again, why did he?” | PCP34-II03 | F, 59 yo, NS |
| Identification of relevant factors | Need regarding nicotine replacement therapy | 10 | “We evaluate the patient’s smoking habits and then we offer him nicotine replacement therapy in line with his smoking habits...” | PCP11-FG02 | M, 36 yo |
| Identification of relevant factors | Current barriers | 6 | “Looking for potential obstacles [to quitting smoking], that’s what it’s all about. […] It’s very individual. The goal is to find and remove all the things that could be conceived of as obstacles...” | PCP16-FG02 | M, 63 yo, NS |
| Identification of relevant factors | Amount of cigarettes smoked during a day with context | 6 | “What I do is to ask them […] to write down, in a small notebook or on a piece of paper, each cigarette they smoke and where they smoke it, with whom, in what circumstances, what they feel, etc...” | PCP33-II02 | F, 34 yo, S |
| Identification of relevant factors | Time of the first cigarette of the day | 3 | “And then we will evaluate how many cigarettes he smokes per day. The first cigarette, at what time does he smoke it after he wakes up…” | PCP34-II03 | F, 59 yo, NS |
| Barriers to action from PCP | Lack of time | 13 | “But if I do everything in 15 minutes, I screw it up. Patients are in a hurry. Excuse me for being vulgar and using the words “screw it up,” but it’s the case.” | PCP01-FG01 | M, 60 yo, S |
| Barriers to action from PCP | Patients’ socioeconomic status | 12 | “We know very well that, unfortunately, we are not at all close to fixing the problem. Even we don’t talk about it a lot, in disadvantaged socio-economic areas, getting the message across [about quitting smoking] is necessarily more complex.” | PCP30-FG04 | M, 42 yo, FS |
| Barriers to action from PCP | Patients’ social environment | 12 | “From my point of view, I see that in families where the parents smoke, the children smoke, save a few exceptions here and there. In families where the parents don’t smoke, the children don’t smoke [either], save a few exceptions.” | PCP18-FG02 | M, 65 yo, S |
| Barriers to action from PCP | Weight gain | 10 | “There is also a big pitfall that I have [seen], people tell me about it and I observe that it’s true, it’s the weight gain when people stop smoking.” | PCP14-FG02 | M, 56 yo, NS |
| Barriers to action from PCP | Fear of withdrawal symptoms | 10 | “We talk about fears related to stopping [smoking]. It’s often weight gain, it’s often the fear of being irritable, […] the fear of starting smoking again.” | PCP34-II03 | F, 59 yo, NS |
| Barriers to action from PCP | Co-occurring addiction | 10 | “When they [patients] come [to an appointment] for another problem, when they come for an alcohol problem, a cannabis problem, obviously it’s not as easy to talk to them about stopping smoking.” | PCP27-FG04 | M, 69 yo, FS |
| Barriers to action from PCP | Patient feeling of invulnerability | 8 | “They don’t care about their health. They are 25, they think [health concerns] are for old people. […] Health for them is not meaningful…they are invincible.” | PCP15-FG02 | M, 45 yo, NS |
| Barriers to action from PCP | Personal lifestyle choice | 7 | “There are people who tell you: anyhow, I don’t intend to live until I’ve 95 years old, I don’t care, might as well die from this [smoking] instead of something else...” | PCP32-II01 | M, 57 yo, FS |
| Barriers to action from PCP | Competing priorities | 6 | “[Some patients have] a job that makes them very tired, we are not there to pester them with questions about tobacco. We will talk about that another time. So, there are priorities.” | PCP19-FG03 | F, 47 yo, NS |
| Barriers to action from PCP | Marketing within the tobacco industry | 5 | “There are industries that developed, really an art so that … it’s a turn-on too […] at that time, I think that in order to also seduce women a little [to buy cigarettes] there were these little thin cigarette packs with very thin cigarettes which actually I think maybe gave a certain attitude, even an almost elegant attitude” | PCP34-II03 | F, 59 yo, NS |
| Barriers to action from PCP | Patient refusal | 5 | “I’m thinking about a patient who had kidney cancer and who smoked two packs a day. I tried to suggest to him that his smoking had something to do with it. He was in denial.” | PCP26-FG04 | F, 70 yo, NS |
| Barriers to action from PCP | PCP feeling illegitimate (as smoker) | 5 | “It’s at once a benefit and maybe also a disadvantage […] We [doctors] sometimes do not have the sufficient authority […] I’m not a smoker’s friend… I’m a doctor. So, I need to sometimes look at things differently when it comes to my empathy which could be a little bit more invasive.” | PCP04-FG01 | F, 55 yo, FS |
| Barriers to action from PCP | PCP feeling illegitimate (as non-smoker) | 4 | “As a non-smoker myself, I need to make an effort to be empathetic so that I can accept that a patient has become addicted to it. Because I have trouble understanding that people become addicted to tobacco because I don't know what it is.” | PCP02-FG01 | F, 47 yo, NS |
| Barriers to action from PCP | Patient feeling overconfident | 3 | “[The patient says to himself] I stop [smoking] like that, it's easy. And then when we look at it together, [I ask the patient] how long did you stop? [The patient says] well, it's been a week, or a month... It's not that easy to make them realize that they have to decide, and get help.” | PCP35-II04 | F, 62 yo, FS |
| Barriers to action from PCP | Lacking skills | 3 | “Smokers with thirty years of smoking and other addictions who have a very particular psychological profile, [...] well, I don't know how to do handle that situation, I'm not used to it and... and then I think it's beyond my skill set.” | PCP33-II02 | F, 34 yo, S |
| Barriers to action from PCP | PCP fear of jeopardizing the therapeutic alliance | 3 | “From the moment a patient comes to see you but not necessarily for that reason [to stop smoking], and you insist on it, you generally do not see them again. There are other doctors he can pay to see and there is no difficulty in finding another doctor. […] But if you insist too much, you don't see the patient again.” | PCP32-II01 | M, 57 yo, FS |
| Barriers to action from PCP | Psychiatric patients | 3 | “When people with psychotic symptoms smoke, you can't stop them.” | PCP35-II04 | F, 62 yo, FS |
| Barriers to action from PCP | Challenging to catch adolescent | 3 | “So we have a lot less at that age. [...] When he [the patient] starts to have frequent bronchitis, we say to ourselves, “well, this patient started smoking.” And so we bring up the subject. Either he says yes or he says no. In fact, when the parents are present I bring it up by saying, “Look, you may not tell me, but beware anyway...” We always try.” | PCP25-FG03 | F, 58 yo, NS |
| Resources | Other healthcare professionals | 25 | “For a long time I was like you and then I managed to prescribe [nicotine replacement therapy] more easily because I rely a lot on a partner that can really help me as a back-up: and that’s pharmacists.” | PCP04-FG01 | F, 55 yo, FS |
| Resources | Websites and smartphone applications | 22 | “As long as you know how to use the app, you're just going to download this app! […] Frankly, the app is indeed an assistant […]. I like it. I find that it works a lot more than the other things! The app is really very modern!” | PCP11-FG02 | M, 36 yo, NS |
| Resources | Personal experience of tobacco smoking cessation | 12 | “I was in my position of an ex-smoker, it was quite normal at that time. What I mean to say is, when there is a risk of starting to smoke again, or if they start smoking again, it’s at that time that I will talk about my own experience, when it was hard to keep going and that I understand how it is for them…I use my own experience.” | PCP05-FG01 | M, 45 yo, FS |
| Resources | Training courses | 10 | “I have attended several trainings about tobacco.” | PCP04-FG01 | F, 55 yo, FS |
| Resources | Patients’ motivation | 9 | “These patients, I tell them that the motivation that matters is not mine, it must come from them! It's not because I'm motivated that you will stop smoking that it will work.” | PCP02-FG01 | F, 47 yo, NS |
| Resources | Pregnancy | 9 | “For women who want to get pregnant, that can be a very big motivator.” | PCP31-FG04 | M, 69 yo, NS |
| Resources | Tobacco smoking denormalization | 9 | “Yes, the norm is not to be a smoker. That is, smoking becomes stigmatized. But it is still something…it is still something that does good.” | PCP23-FG03 | M, 35 yo, FS |
| Resources | Full reimbursement of most nicotine replacement therapy | 8 | “A reimbursed [nicotine] patch is a medical drug, it’s a medical drug that can be prescribed by a doctor. Go see the doctor to have him reimburse your patch. It’s the chance to see him for a real appointment about stopping tobacco.” | PCP13-FG02 | M, 60 yo, FS |
| Resources | Events such as “Stoptober” | 7 | “What works is the month without tobacco [a national event in France each year]; sometimes it’s a type of challenge for them to stop, I find that it works well.” | PCP32-II01 | M, 57 yo, FS |
| Resources | Price increase of cigarettes | 5 | “The argument that works for stopping smoking is an aggressive increase in cost, not a progressive increase in cost” | PCP13-FG02 | M, 60 yo, FS |
| Resources | Printed materials (flyers, posters) | 5 | “I think that what is important is the importance of non-verbal communication and the posters that can be found in doctors’ waiting rooms. […] In order to say to the patient: you can talk about this topic with your general practitioner.” | PCP31-FG04 | M, 69 yo, NS |
| Resources | Medical research progress | 3 | “I was a little shocked at the last few studies I read on the, the importance of the length of impregnation versus the amount smoked. According to what we were taught in university in fourth year, it's completely outdated! Now, it is really the duration of impregnation which is the most important. […] By definition, we are supposed to change our arguments according to scientific advances.” | PCP13-FG02 | M, 60 yo, FS |
| Resources | Assessment tools | 3 | “It’s a test that measures carbon monoxide […] : it’s a way to see how much smoke he has inhaled.” | PCP34-II03 | F, 59 yo, NS |
| Resources | Child passive tobacco smoking | 3 | “When it comes to babies, that’s a chance to insist on passive smoking.” | PCP23-FG03 | M, 35 yo, FS |
| Treatment and interventions | Serious adverse events of varenicline | 7 | “I can also prescribe them varenicline sometimes but more as a second option and the electronic cigarette quite often, too, but always alongside the [nicotine] patches. Like the electronic cigarette for me instead of the gum or the thing to suck on a little bit.” | PCP13-FG02 | M, 60 yo, FS |
| Treatment and interventions | Serious adverse events of varenicline | 7 | “It [varenicline] has a bad reputation... Because there are psychiatric side effects actually.” | PCP26-FG04 | F, 70 yo, NS |
| Treatment and interventions | Supporting therapy | 29 | “So, in general, I give them the contact details of a certain number of specialized colleagues who do either acupuncture, auriculotherapy, or homeopathy.” | PCP32-II01 | M, 57 yo, FS |
